# External Ventricular Drain Infections: Risk Factors and Outcome

**DOI:** 10.1155/2014/708531

**Published:** 2014-11-17

**Authors:** S. Hagel, T. Bruns, M. W. Pletz, C. Engel, R. Kalff, C. Ewald

**Affiliations:** ^1^Center for Infectious Diseases and Infection Control, Jena University Hospital, Jena, Germany; ^2^Center for Sepsis Control & Care (CSCC), Jena University Hospital, Jena, Germany; ^3^Department of Internal Medicine IV, Gastroenterology, Hepatology and Infectious Diseases, Jena University Hospital, Jena, Germany; ^4^Institute of Medical Informatics, Statistics and Epidemiology, University of Leipzig, Leipzig, Germany; ^5^Department of Neurosurgery, Jena University Hospital, Jena, Germany

## Abstract

External ventricular drainage (EVD) is frequently used in neurosurgery to drain cerebrospinal fluid in patients with raised intracranial pressure. We performed a retrospective single center study in order to evaluate the incidence of EVD-related infections and to identify underlying risk factors. 246 EVDs were placed in 218 patients over a 30-month period. EVD was continued in median for 7 days (range 1–44). The cumulative incidence of EVD-related infections was 8.3% (95% CI, 5.3–12.7) with a device-associated infection rate of 10.4 per 1000 drainage days (95% CI, 6.2–16.5). The pathogens most commonly identified were coagulase-negative* Staphylococcus* (62%) followed by* Enterococcus* spp. (19%). Patients with an EVD-related infection had a significantly longer ICU (11 versus 21 days, *P* < 0.01) and hospital stay (20 versus 28.5 days, *P* < 0.01) than patients without. Median total duration of external drainage was twice as long in patients with EVD-related infection (6 versus 12 days, *P* < 0.01). However, there was no significant difference in the duration between first EVD placement and the occurrence of EVD-related infection and EVD removal in patients without EVD-related infection (6 versus 7 days, *P* = 0.87), respectively. Interestingly no risk factor for EVD-related infection could be identified in our cohort of patients.

## 1. Introduction

Ventriculostomy catheters, also known as external ventricular drains (EVDs), are frequently used in neurosurgery to monitor and relief intracranial pressure. Complications arising from EVDs include hemorrhage, misplacement, dislodgement, blockage, and, most significantly, infection, which may be complicated by ventriculitis, meningitis, brain abscess, or subdural empyema. EVD-related infections significantly prolong hospital stay, increase costs, and often negatively affect the overall prognosis. Reported rates of EVD-related infections range from <5% up to 23%, most commonly close to 10% [1]. An increased risk of infection has been observed in patients with subarachnoid or intraventricular hemorrhage, in patients with concurrent systemic infections as well as with longer duration of catheterization, cerebrospinal (CSF) leakage, and frequent manipulation of the EVD system [2–4]. In addition we hypothesized that multibed accommodation of patients with EVD in a mixed surgical intensive care setting may constitute a risk factor for EVD-related infections. The objective of the present study was to assess the incidence and outcome of EVD-related infections and to identify new and confirm already known risk factors.

## 2. Methods

For this single-center retrospective study we included all patients over 18 years of age that underwent EVD placement during a 30-month period (January 2010 to June 2012) at our 1.500 bed tertiary center (Jena University Hospital, Germany). For drainage conventional (Promedics) and silver-impregnated catheter (VentriGuard) were used. All catheters were inserted under sterile conditions in the operating theatre using a tunneled procedure technique and a closed system for drainage. There was no policy of routine CSF sampling or replacement of catheters during the whole study period. The third-generation cephalosporin Ceftriaxone was given at 2 g once daily to all patients from insertion until EVD removal.

Patients that were not immediately transferred to the ICU after EVD insertion (*n* = 5), patients presenting with open skull fracture and CSF leakage (*n* = 4), and patients with active infection of the central nervous system (CNS) at first EVD implantation (*n* = 18) were excluded from the study. A total of 218 patients were included in the study. The 26-bed ICU is a mixed ICU treating patients of all surgical disciplines in four single-bed rooms, seven two-bed, and two four-bed rooms. Patients were followed up for 7 days after discharge from ICU. The charts of all patients were retrospectively reviewed and demographics, ASA-score (physical status classification of preoperative patients for anaesthetic risk assessment from the American Society of Anaesthesiologists), EVD-related data, type of accommodation, and underlying or arising healthcare-associated infections during ventricular drainage were documented. The study was approved by the institutional review board.

### 2.1. Definition of Infection

We defined EVD-related infection as (1) positive CSF culture result* plus* clinical symptoms* or* CNS pleocytosis/cell count increase, or (2) in the case of negative CSF culture, clinical symptoms,* and* CNS pleocytosis/cell count increase [5, 6]. Healthcare-associated infections were defined using the CDC/NHSN surveillance definitions [7].

### 2.2. Statistical Analysis

Continuous values were expressed as median (range) and compared using the Mann-Whitney *U* Test. Categorical data were presented as frequencies and percentages and compared using the chi-square test or Fisher's exact test where appropriate. Cox regression analysis and the Kaplan-Maier method were used to determine predictors of EVD-related infection. On the basis of Poisson distribution and Wilson-Score method, respectively, we calculated the confidence intervals for the incidence density and cumulative incidence. *P* values <0.05 in two-sided testing were considered significant. All analyses were performed using IBM SPSS Statistics version 22.

## 3. Results

Two hundred and forty-six EVDs were placed in 218 patients. External drainage was continued in median for 7 days (range 1–44) resulting in 1.725 catheter days (Table 1). The first and second EVD remained each in place for a median of 6 days (1st: range 1–19 days, 2nd: range 1–17 days), the third EVD for 12 days (range 9–16). One patient required a fourth and fifth EVD which remained in situ for 11 and 12 days, respectively. Indications for extraventricular drainage were nontraumatic subarachnoid and/or intraventricular hemorrhage in 133 (61%) patients, traumatic brain injury with subarachnoid and/or intraventricular hemorrhage in 38 (17%) patients, intracranial tumors in 25 (12%) patients (*n* = 8 benign; *n* = 17 malignant), and others in 22 (10%) patients (*n* = 16 contusion/edema; *n* = 6 hydrocephalus).

Eighteen patients developed an EVD-related infection resulting in a cumulative incidence of 8.3% (95% CI, 5.3–12.7). All patients experienced only one episode of infection. In 14 patients the first EVD was infected, in four patients the second one. Six patients with an infection of the first EVD received a second EVD, one of those a third, and one patient five EVDs in total. Reasons for a change were the underlying infection and/or clogging with still necessity of ventricular drainage. Thirteen patients without an EVD-related infection received a second EVD; the reasons were displacement or clogging.

**Figure 1:**
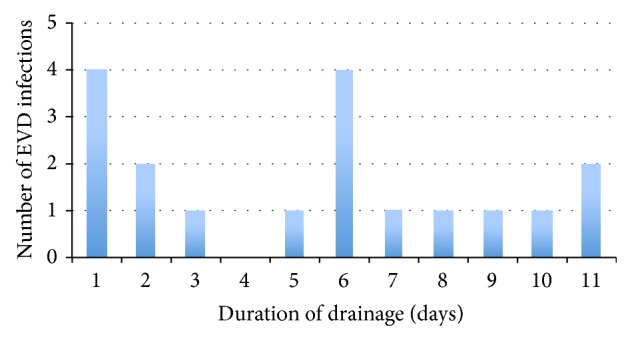
Occurrence of EVD-related infection related to drainage days after placement of EVD with underlying infection.

The device-associated infection rate was 10.4 per 1.000 (95% CI, 6.2–16.5) EVD days considering the total time of EVDs in place (1.725 days) and 11.8 per 1000 (95% CI, 6.9–18.6) EVD days considering catheter days without a previous EVD-related infection (1.572 days). On average an EVD-related infection became in mean evident 6 days (range 1–11) after insertion. Seven EVD-related infections were diagnosed within three days after EVD placement (Figure 1). In 88 EVD procedures a conventional catheter was used and in 122 procedures a silver-impregnated catheter was inserted. In 26 procedures the type of the used catheter was not documented. No significant difference of EVD-related infection rate was found between patients with conventional EVD or silver-impregnated EVD (2% versus 9%; *P* = 0.08). Of 18 EVD infections, 16 (89%) were proven based on microbiological test results. No polymicrobial infection was found. The pathogens most commonly identified were coagulase-negative* Staphylococcus* (CNS) (62%) followed by* Enterococcus* spp. (19%) and other pathogens (19%) including* Klebsiella pneumoniae, Escherichia coli,* and* Micrococcus luteus* (Table 2).

No association of EVD-related infections with demographical parameters, indication for EVD placement, and the type of accommodation could be detected (Table 3). Furthermore, there was no difference in mortality (single-bed 14% versus multibed 18%, *P* = 1.0) or in the occurrence of healthcare-associated infections other than EVD-related infection between patients accommodated in single-bed rooms and multiple-bed rooms, respectively (21% versus 25%, *P* = 1.0). Concomitant healthcare-associated infections (HAIs) were found significantly more often in patients with EVD-related infections (44% versus 23%, *P* < 0.01), with a trend towards more surgical site infections (SSI), urinary tract infections (UTI), and central line-associated bloodstream infections (CLABSI) in patients with EVD-related infections. No coherence between the organisms responsible for HAIs and those responsible for the EVD-related infection was found. Patients with an EVD-related infection had a significantly longer ICU (11 versus 21 days, *P* < 0.01) and hospital stay (20 versus 28.5 days, *P* < 0.01) than patients without. Median total duration of external drainage was twice as long in patients with EVD-related infection (6 versus 12 days, *P* < 0.01). However, there was no significant difference in the duration between first EVD placement and the occurrence of EVD-related infection, respectively, EVD removal in patients without EVD-related infection (6 versus 7 days, *P* = 0.87). When controlled for the duration of the infection-free EVD drainage as a continuous variable, the occurrence of any HAI remained a statistically significant risk factor for the development of an EVD-related infection in a multivariate logistic regression model (univariate OR 4.3, 95% CI 1.6–11.6; adjusted OR 7.1, 95% CI 2.2–22.9), whereas the duration of drainage until infection or removal was not. Cox regression analysis did not identify a significant association between any further studied parameter and EVD-related infection. A change of an EVD before infection or removal of the EVD was also not associated with an increased risk for a subsequent EVD-related infection (*P* = 0,47). EVD-related infection was not associated with increased in-hospital mortality (17% versus 18%; *P* = 1.0) The only parameter associated with adverse outcome was ASA-score classification with an odds ratio of 2.7 per 1-class increase (*P* < 0.01).

## 4. Discussion

The cumulative incidence of EVD-related infection in our study was 8.3% and hence comparable with previous published studies [1–3]. The device-associated infection rate observed in this study (10.4 per 1000 EVD-days) is higher than the rate reported by Scheithauer et al. (6.3 per 1000 device days), which has been the only device-associated infection rate reported until now [5]. Noticeably, the mean duration of drainage time in patients without EVD-related infection in that study was considerably longer compared to our cohort (11 versus 8 days). The majority of published series reported a statistically significant higher incidence of EVD-related infections in patients with subarachnoid and/or intraventricular hemorrhage when compared with patients with nonhemorrhagic pathologies [1, 4]. According to two previous studies [6, 7] we however could not confirm subarachnoid and/or intraventricular hemorrhage as an independent risk factor for EVD-related infection in logistic regression analysis. Reasons for that discrepancy are not clear. Several studies examined the relationship between concurrent systemic infections and EVD-related infection, showing that concurrent systemic infections are a risk factor for EVD-related infection [1]. These results could not be confirmed in our study, in which the presence of a healthcare-associated infection during external ventricular drainage was not a risk factor for EVD-related infection. However, patients with an EVD-associated infection had significantly more often concurrent healthcare-associated infections, which might be a consequence of the prolonged length of stay in the intensive care unit. As in previous studies there was no coherence between the organism responsible for the healthcare-associated infection and the organism responsible for the EVD-related infection in the individual patient [1].

Multiple studies examined the duration of catheterization as a risk factor for EVD-related infection. Interpretation of these studies is complicated because some investigators used cumulative infection rates, either uncorrected or censored by the use of life-table analysis, while others used daily infection rates. Even though there is some controversy regarding the actual daily infection rate, Lozier et al. [1] could show that the hazard rate varies over time, suggesting daily changing infection risks. Although the median total duration of external drainage was twice as long in patients with EVD-related infection than in patients without infection, there was no difference between insertion of the first EVD and occurrence of EVD-related infection or EVD removal in patients without EVD-related infection. This underlines the fact that prolongation of total drainage time in patients with EVD infection is a result of EVD infection and not vice versa. In line with these findings seven EVD-related infections were diagnosed within three days after placement of the infected EVD. Four of the seven EVD-related early infections were caused by coagulase-negative* Staphylococci* (CNS) which may have arisen from initial inoculations, which develop in detectable infections after variable incubation periods of around five days as previously suggested [8]. This emphasizes the necessity of EVD placement under fastidious aseptic conditions. In accordance with previous studies, coagulase-negative* Staphylococci* were the bacteria most commonly isolated in patients with EVD-related infections accounting for 62% of cases. Other common organisms include* Enterococcus* spp.,* Enterobacter* spp., and* Staphylococcus aureus *[9]. This pattern coincides with that of the usual skin flora and hospital environment. Concerning our additional research question, whether multibed accommodation of patients in the ICU setting poses a risk factor for EVD-related infections we were not able to show any difference in the infection rate between patients who were placed in single- or multibed rooms, respectively.

The current notion is that EVD-related infections result from either inoculation of pathogens during EVD placement and/or contamination and colonization of the EVD system during the postoperative period [1]. Postoperative colonization can either arise from endogenous organisms present on the skin, which spread along the intracutaneous tract or by exogenous organisms introduced into the EVD system during manipulation at the EVD system by healthcare workers. Endogenous infections might be prevented by using antimicrobial coated EVD catheters which may decrease bacterial colonization and thus prevent infection. Just recently Wang et al. [10] performed a meta-analysis to assess the efficacy of antimicrobial-impregnated catheters in preventing catheter-related infections during external ventricular drainage. Compared with standard catheters, a significantly lower rate of CSF infection was noticed for clindamycin/rifampin-impregnated catheters (OR 0.27, 95% CI, 0.10–0.73, *P* < 0.05) and for minocycline/rifampin-impregnated catheters (OR 0.11, 95% CI, 0.06–0.21, *P* < 0.05). No statistical significance was found when standard catheters were compared with silver-impregnated catheters (OR 0.33, 95% CI = 0.07 to 1.69, *P* = 0.18). In the meantime however several additional studies were published which assessed the efficacy of silver-impregnated catheters. Winkler et al. [11] compared in their prospective randomized trial the rate of EVD-related infections of 61 EVD placements with either antibiotic-coated (*n* = 32) or silver-bearing catheters (*n* = 29) in 40 patients. Regarding CSF infection rate and dysfunction, no statistical significant differences between the two EVD catheters Bactiseal versus VentriGuard were found. Lajcak et al. [12] performed a retrospective study of 403 patients with a total of 529 implanted EVDs. The rate of infections by catheter type was 7.6% (11/145) and 13.8% (4/29) for two different types of noncoated polyurethane catheters. Silver-impregnated polyurethane catheters became infected in 6.1% (14 out of 228). The differences between noncoated and silver-coated catheters were statistically significant. Keong et al. [13] performed a randomized controlled trail in overall 278 patients. There was a significant difference in infection risk between the two study arms: 21.4% (30/140) for plain catheters versus 12.3% (17/138) for silver catheters (*P* = 0.04). In contrast to these studies, however, we could show a trend towards a higher infection rate in patients with a silver-coated EVD catheter when compared to patients with nonimpregnated catheters (9% versus 2.3%; *P* = 0.08). The reason for this observation is not obvious. Selection bias can be excluded as the catheters were not available in our institution at the same time.

### 4.1. Study Limitations

Because of the retrospective nature of the present study, various limitations must be mentioned. Particularly, data collection is compromised by missing values. Furthermore our findings might be limited due to the number of patients with EVD-related infection which was smaller than infected and the small number of patients who were accommodated in single-bed rooms.

## 5. Conclusion

Many studies have been conducted to identify risk factors of EVD-related infections. However, none of these risk factors could be confirmed in our cohort of patients. Furthermore we could not show any difference in infection rates between patients who were placed in single- or multibed rooms, respectively.

## Figures and Tables

**Table 1:** EVD catheter data.

EVD	Patients (*n*)	Catheter days (*n*)
1st EVD	218	1515
2nd EVD	23	150
3rd EVD	3	37
4th EVD	1	11
5th EVD	1	12

**Table 2:** Microbial culture results of episodes of EVD infection.

Organism	*n* (day of CSF sampling in current EVD)
*Enterococcus *spp.	
*E. faecium *	2 (day 1, 3)
Unclassified	1 (day 6)
*Klebsiella pneumoniae *	1 (day 5)
*Staphylococcus *spp.	
*Staphylococcus hominis *	1 (day 10)
*Staphylococcus capitis *	1 (day 11)
*Staphylococcus haemolyticus *	1 (day 6)
*Staphylococcus epidermidis *	7 (day 1, 1, 2, 2, 6, 8, 11)
*E. coli *	1 (day 7)
*Micrococcus luteus *	1 (day 6)

**Table 3:** Patient demographics and outcome of EVD-related infections.

	No EVD infection (*n* = 200)	EVD infection (*n* = 18)	*P*
Sex, *n* (%)			0.46
Male	111 (93%)	8 (7%)	
Female	89 (90%)	10 (10%)	
Age (years)	61 (18–86)	51 (18–81)	0.13
BMI	25.5 (18–45)	25.5 (18–48)	0.65
ASA-score, *n* (%)			0.46
1	7 (4%)	0 (0%)	
2	40 (20%)	2 (11%)	
3	87 (43%)	11 (61%)	
4	66 (33%)	5 (28%)	
Admission, diagnosis, *n* (%)			0.54
SAH, nontraumatic	120 (60%)	13 (71%)	
SAH, traumatic	36 (18%)	2 (11%)	
Tumour, malignant	17 (8%)	0 (0%)	
Tumour, benign	7 (4%)	1 (6%)	
Hydrocephalus	5 (2%)	1 (6%)	
Contusion/oedema	15 (8%)	1 (6%)	
History, *n* (%)			
Cancer, haematology	6 (3%)	0 (0%)	0.45
Cancer, solid	18 (9%)	1 (6%)	0.62
Diabetes mellitus	31 (16%)	1 (6%)	0.25
Immunosuppressive	2 (1%)	0 (0%)	0.67
Neurosurgical procedure (besides of EVD placement)	42 (21%)	3 (17%)	1.0
EVD-procedure, *n* (%)			0.91
Elective	24 (12%)	2 (11%)	
Emergency	176 (88%)	16 (89%)	
Accommodation, *n* (%)			0.50
Single-bed room	12 (6%)	2 (11%)	
Double-bed room	93 (46%)	7 (39%)	
Four-bed room	95 (48%)	9 (50%)	
Concomitant infection, *n* (%)			
ANY	45 (23%)	8 (44%)	<0.01
LRTI	42 (21%)	6 (33%)	0.23
SSI	1 (1%)	2 (11%)	0.02
UTI	0 (0%)	1 (6%)	0.08
CLABSI	2 (1%)	2 (11%)	0.05
Other	1 (1%)	0 (0%)	1.0
In-hospital death, *n* (%)	36 (18%)	3 (17%)	1.0
External drainage, (total duration), days	6 (1–20)	12 (4–44)	<0.01
External drainage, (total duration of infection-free drainage)	6 (1–20)	7 (1–16)	0.87
LOS-hospital, days	20 (1–90)	28.5 (15–74)	<0.01
LOS-ICU, days	11 (0–76)	21 (10–50)	<0.01

SAH: subarachnoid hemorrhage, ASA: American Society of Anesthesiologists, LRTI: lower respiratory tract infection, SSI: surgical site infection, UTI: urinary tract infection, CLABSI: central line- associated bloodstream infection, LOS: length of stay.
